# Effect of Slow Versus Rapid Advancement of Enteral Feeding on Intestinal Oxygenation in Preterm Infants

**DOI:** 10.3390/children12111527

**Published:** 2025-11-11

**Authors:** Hulya Ozdemir, Sinem Gulcan Kersin, Halime Sema Can Buker, Merih Cetinkaya, Ibrahim Kandemir, Asli Memisoglu, Hulya Selva Bilgen

**Affiliations:** 1 Department of Pediatrics, Division of Neonatology, Marmara University Pendik Training and Research Hospital, Istanbul 34899, Türkiye; 2 Department of Neonatology, Başakşehir Çam and Sakura City Hospital, Istanbul 34480, Türkiye; 3 Department of Pediatrics, Istanbul Health and Technology University, Istanbul 34270, Türkiye

**Keywords:** preterm infant, intestinal oxygenation, enteral feeding, slow, rapid advancement, feeding intolerance

## Abstract

**Highlights:**

**What are the main findings?**

- Intermittent bolus feeding increased intestinal oxygenation, with a more pronounced percentage change observed during and after feeding in the rapid advancement group.
- No significant difference in gastrointestinal adverse outcomes, such as feeding intolerance or necrotizing enterocolitis, was found between the slow and rapid advancement groups.
- The use of abdominal near-infrared spectroscopy allowed continuous, real-time assessment of mesenteric oxygenation, demonstrating its potential value as a non-invasive monitoring tool in preterm infants.

**What is the implication of the main finding?**

- Rapid advancement of enteral feeding, when closely monitored clinically and/or with abdominal near-infrared spectroscopy, may be safely implemented without increasing the risk of gastrointestinal complications.

**Abstract:**

**Background/Objectives:** The optimal rate of enteral feeding advancement in preterm infants remains uncertain despite decades of clinical research. This uncertainty arises from concerns that rapid feeding progression may increase the risk of feeding intolerance and necrotizing enterocolitis (NEC), two major causes of morbidity and mortality in this population. The feeding rate may also influence intestinal oxygenation due to mesenteric hemodynamic changes during feeding. This study aimed to evaluate whether the rate of enteral feeding advancement (slow vs. rapid) affects intestinal oxygenation and its association with feeding intolerance (FI) or necrotizing enterocolitis in very low birth weight preterm infants. **Methods:** This prospective, randomized, two-center study included infants born at 28–32 weeks of gestation. Group 1 received slow advancement (20 mL/kg/day) and Group 2 rapid advancement (30 mL/kg/day) of enteral feeds. Splanchnic (srSO_2_) and cerebral (crSO_2_) oxygenation were monitored daily using the FDA-approved INVOS NIRS device during feeding periods (08:00–16:00). Monitoring was performed during minimal enteral nutrition (Phase 1), advancement phases (Phase 2), and for two days after achieving full enteral feeding (Phase 3). The splanchnic-to-cerebral oxygenation ratio (SCOR) was also calculated. Percentage changes in srSO_2_ and SCOR during and after feeding were calculated from baseline (prefeeding) values and analyzed. **Results:** Sixty infants were enrolled. Mean gestational age and birth weight were 29.76 ± 1.33 weeks and 1375.05 ± 271.19 g, respectively. Group 2 achieved full enteral feeding significantly earlier (*p* = 0.001), with no other demographic differences between groups. No cases of NEC were observed. Feeding intolerance occurred in 14 infants (23.3%): 8 in Group 1 and 6 in Group 2 (*p* = 0.192). Both groups exhibited increased srSO_2_ and SCOR during feeding; however, the between-group differences were not statistically significant (Phase 2 srSO_2_ and SCOR: *p* = 0.07, 0.08; Phase 3 srSO_2_ and SCOR: *p* = 0.069, 0.071). However, the percentage change from baseline in srSO_2_ and SCOR during and after feeding was significantly greater in Group 2 during the advancement and full enteral feeding phases (Phase 2 srSO_2_ and SCOR: *p* = 0.03, 0.022; Phase 3 srSO_2_ and SCOR: *p* = 0.015, 0.048). Infants with feeding intolerance demonstrated significantly lower srSO_2_ and SCOR values compared to tolerant infants, and this reduction persisted even after reaching full enteral feeding. ROC analysis suggested gestational age < 30 weeks, birth weight < 1180 g, srSO_2_ < 52, and SCOR < 0.6 were associated with feeding intolerance. **Conclusions**: Intermittent bolus feeding increased intestinal oxygenation, with a more pronounced effect in the rapid advancement group. No difference in gastrointestinal adverse outcomes was observed between groups. Lower intestinal oxygenation was associated with feeding intolerance, and the suggested predictive criteria may help guide individualized feeding strategies.

## 1. Introduction

There is an ongoing debate regarding the appropriate timing for initiating feeding and the rate at which feeding volumes should be advanced [1,2,3,4]. In this context, there is no consensus on best practices for enteral nutrition in preterm infants, and the question of whether feeding volumes should be advanced rapidly or slowly continues to be controversial [5,6,7]. Significant variability exists across clinical settings in both the timing of feeding initiation and the progression rate of enteral feeding [1,2,3,5,8,9]. A recent Cochrane meta-analysis reported, with moderate-certainty evidence, that rapid advancement does not increase the risk of necrotizing enterocolitis (NEC) [2]. In light of these findings, the progression of enteral feeding remains an essential yet debated aspect of managing hospitalized preterm and very low birth weight infants. Clinicians have often preferred gradual increases in milk volume due to concerns about feeding intolerance and NEC. Earlier observational studies suggested that slower advancement may be associated with a lower risk of developing NEC [8,10,11,12,13]. On the other hand, gradually advancing feeds comes with potential drawbacks. It extends the duration of parenteral nutrition, increasing the likelihood of complications such as liver disease related to parenteral nutrition and bloodstream infections linked to central lines [4,14]. Preterm and very low birth weight infants are physiologically immature and therefore at high risk for feeding intolerance and necrotizing enterocolitis; thus, careful advancement of enteral feeding is of great importance [15]. Although various feeding regimens have been developed for these infants, practices often vary according to local experience and tradition, and related discussions and studies in the literature are ongoing.

The determinants of how enteral feeding influences intestinal perfusion in preterm infants have yet to be comprehensively studied [16]. Studies indicate that the introduction of enteral feeds increases the small intestine’s metabolic requirements, leading to greater blood flow from the superior mesenteric artery (SMA), a process known as postprandial hyperemia [17,18]. Using near-infrared spectroscopy (NIRS), a non-invasive method, it is possible to assess end-organ perfusion in preterm infants by continuously measuring regional tissue oxygen saturation (rSO_2_) [19]. Recent findings from studies on NIRS and Doppler flow in the superior mesenteric artery (SMA) indicate that increased postprandial intestinal perfusion without changes in cerebral perfusion [20,21,22]. NIRS, a non-invasive method for assessing end-organ blood flow and continuously measuring regional oxygen saturation (rSO_2_), may therefore help monitor mesenteric perfusion in preterm infants at risk of gastrointestinal complications such as necrotizing enterocolitis (NEC) [20,21,22].

In this context, this study aimed to evaluate the effects of rapid versus slow advancement of enteral feeding on intestinal oxygenation and their association with feeding intolerance and necrotizing enterocolitis in very low birth weight preterm infants.

## 2. Materials and Methods

### 2.1. Subjects

This randomized, prospective, two-center clinical trial included neonates admitted to the NICUs of Marmara University Pendik Training and Research Hospital and Başakşehir Çam and Sakura City Hospital between October 2021 and September 2022. This prospective interventional study was conducted in accordance with the ethical principles outlined in the Declaration of Helsinki and its subsequent revisions. The study protocol and informed consent forms were reviewed and approved by the Ethics Committee of Marmara University Pendik Training and Research Hospital (Approval No: 09.2021.612). Written informed consent was obtained from the parents or legal guardians of all participating infants prior to enrollment. Participation in the study was entirely voluntary, and families retained the right to withdraw from the study at any time without affecting the standard clinical care of their infants. To ensure confidentiality, all data were anonymized prior to analysis. The study was registered retrospectively with the Australian New Zealand Clinical Trials Registry (ACTRN12625001045404) (https://anzctr.org.au/ACTRN12625001045404.aspx), accessed on 22 September 2025).

Preterm infants born between 28 and 32 weeks of gestational age were eligible for inclusion in this study. The exclusion criteria consisted of perinatal asphyxia, multiple organ dysfunction, congenital anomalies, intrauterine growth restriction, and impaired skin integrity that prevented the proper placement of NIRS probes.

### 2.2. Randomization

Participants were assigned to study groups using simple randomization, generated through a computer-based random number table. Allocation concealment was ensured via a centralized, automated system that assigned participants immediately after enrollment, preventing foreknowledge of group allocation prior to intervention initiation. Because the intervention involved feeding protocol implementation in preterm infants, blinding of clinical staff was not feasible. Bedside nurses responsible for protocol administration remained unblinded for practical reasons. However, to minimize assessment bias, outcome evaluations were performed by independent investigators who were blinded to group allocation. Therefore, the study employed a single-blind design, maintaining blinding exclusively for independent reviewers.

### 2.3. Intervention

In this study, infants were randomly assigned to two groups: Group 1 received slow enteral feeding advancement at 20 mL/kg/day, while Group 2 received rapid advancement at 30 mL/kg/day. The infants included in the study were initially started on minimal enteral feeding at 10–20 mL/kg/day. After a period of 3 to 5 days, enteral nutrition was advanced according to the study protocol. All infants were fed intermittently via bolus feeding for 10 min. Feeding intervals were set at two hours for infants <1250 g and three hours for those >1250 g. Infants with feeding intolerance were fed using intermittent prolonged infusion, either two hours or one hour.

Feeding intolerance was defined as follows: the withholding of enteral feeding for ≥24 h, required by the inability to digest enteral nutrition, characterized by a gastric residual volume of more than 50%, abdominal distension, vomiting, or a combination of both, along with the disruption of the patient’s feeding plan. In this study, breast milk was the first preferred feeding option, and preterm formula was used for those with inadequate breast milk supply [23].

To assess splanchnic oxygenation (srSO_2_) and cerebral oxygenation (crSO_2_), real-time measurements were conducted using the FDA (Food and Drug Administration) approved INVOS Cerebral/Somatic Oximeter (Covidien, Boulder, CO, USA) NIRS device.

A self-adhesive transducer that contains a light-emitting diode and two distant sensors was placed on the forehead and infra-umbilical abdomen region of patients. All measurements were taken with infants in a supine position. During data recording, infants were mostly quiet or sleeping, and to reduce NIRS artifacts, the handling of patients during the study period was minimized. For the purpose of standardizing data collection, NIRS monitoring was performed once daily, and data were collected during two feeding periods between 08:00 and 16:00 h.

**Figure 1:**
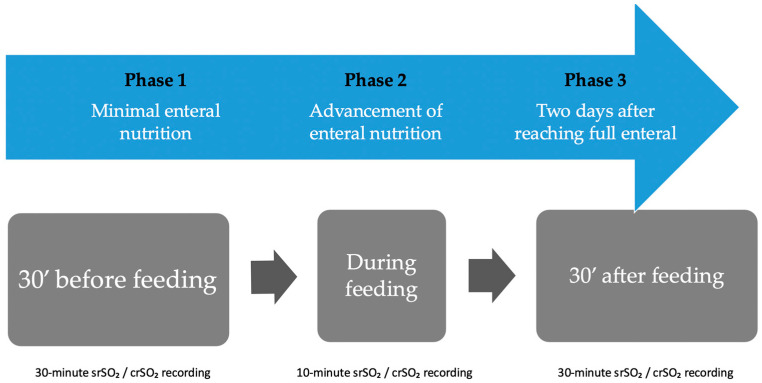
Timing of abdominal NIRS monitoring and definition of feeding phases during the enteral nutrition.

The NIRS monitoring period was divided into three phases: Phase 1, minimal enteral nutrition; Phase 2, advancement of enteral nutrition; and Phase 3, two days after reaching full enteral nutrition (Figure 1).

NIRS measurements were taken 30 min before feeding (baseline), during feeding (10 min), and for 30 min after feeding. NIRS values were recorded at 30 s intervals, and the mean of these values was calculated (Figure 1).

Percentage changes in srSO_2_ and SCOR during and after feeding were calculated from baseline (prefeeding) values in phases 1, 2, and 3. We also calculated the splanchnic-cerebral oxygenation ratio (SCOR, srSO_2_/crSO_2_), the ratio of oxygen saturation of splanchnic versus cerebral tissue. Since cerebral perfusion is subject to autoregulation while splanchnic perfusion is not, SCOR is reduced when there is a diversion of blood flow toward the vital organs, while it remains unchanged under normal conditions.

### 2.4. Sample Size

We used Sample Power version 2 (SPSS Inc., Chicago, IL, USA) to calculate the sample size. On the basis of preliminary data, we determined that we would need 30 cases to have a 90% power to detect a 10% change in SCOR and srSO_2_ from baseline to postprandial measurement (mean difference 0.14, s.d. of the difference 0.23), with an error of 0.05 and a two-tailed paired Student’s *t*-test [24].

### 2.5. Statistical Analysis

Normality was assessed using the Kolmogorov–Smirnov test, kurtosis, skewness, and Q–Q plots. Homogeneity of variances was evaluated using Levene’s test. According to data distribution characteristics, comparisons between two independent groups for continuous variables were performed using Student’s *t*-test when data were normally distributed and variances were homogeneous, Welch’s *t*-test when data were normally distributed but variances were not homogeneous, and the Mann–Whitney *U* test when data were not normally distributed. The chi-square test was used to analyze categorical variables.

To evaluate changes in repeated measures, a Bayesian repeated-measures ANOVA was conducted, and Bayes factors (BF_10_) were reported. For the alternative hypothesis (H_1_), BF_10_ values between 1 and 3 were interpreted as anecdotal evidence, 3–10 as moderate, 10–30 as strong, 30–100 as very strong, and values greater than 100 as extreme evidence. For the null hypothesis (H_0_), BF_10_ values between 0.3 and 1 indicated anecdotal evidence, whereas values between 0.1 and 0.3 indicated moderate evidence.

Receiver operating characteristic (ROC) curves were constructed, and specificity, sensitivity, positive predictive value (PPV), negative predictive value (NPV), and area under the curve (AUC) were calculated. DeLong test and bootstrapping (for 100 runs) were performed to validate the ROC analysis. The DeLong test indicated a significant difference between the novel chart and each of the factors contributing to it. The AUC (of the new chart) with 95% confidence interval was 0.989 (0.973–1.000).

All statistical analyses were conducted using JAMOVI software (version 2.3.18) with the jsq module. The significance level (α) for frequentist analyses was set at 0.05.

## 3. Results

**Figure 2:**
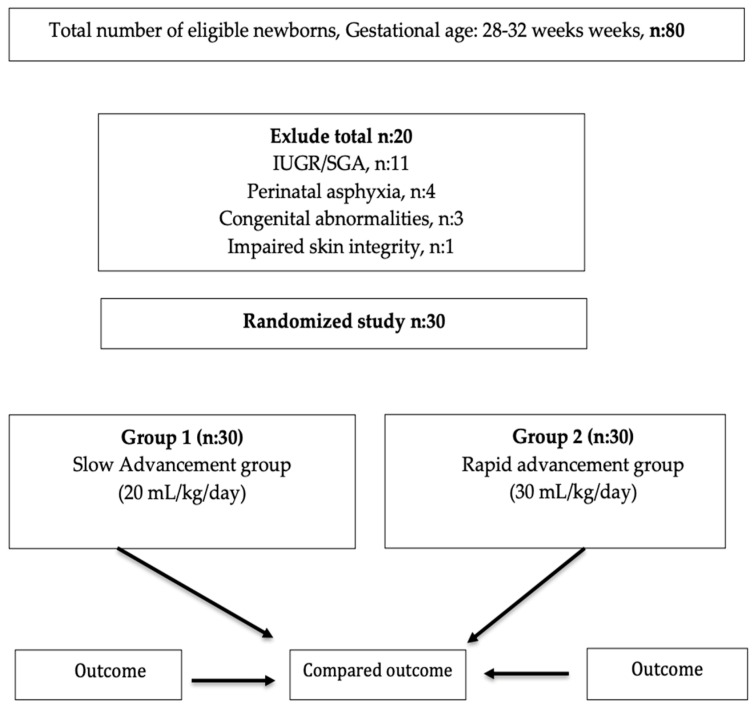
Flow diagram of study population.

During the study period, a total of 80 infants were born between 28 and 32 weeks of gestation. Twenty preterm infants were excluded from the study. A total of 60 infants were included in the study (Figure 2).

The gestational age and birth weight of the infants included in the study were determined as 29.7 ± 1.3 weeks and 1304 ± 253 g, respectively. No statistically significant differences were found in the demographic characteristics of Groups 1 and 2 (Table 1). However, the time to transition to full enteral feeding in Group 2 was significantly shorter than in group 1 (*p* < 0.005) (Table 1).

Feeding intolerance was observed in 14 infants (23.3%). When the groups were compared in terms of the frequency of feeding intolerance, no significant difference was found (Group 1: 8 cases, Group 2: 6 cases, *p* = 0.193). When the demographic and clinical characteristics of infants with and without feeding intolerance were compared, birth weight and gestational age were found to be significantly lower in infants with feeding intolerance (*p* < 0.001). In addition, the duration of minimal enteral nutrition and the time to achieve full enteral feeding were significantly longer in infants with feeding intolerance compared to those who tolerated feeding (*p* < 0.01). No definite NEC was observed in any of the infants.

### 3.1. Evaluation of srSO_2_ and SCOR Alterations in Group 1 and Group 2

The feeding process was evaluated in three phases: minimal enteral nutrition (Phase 1), advancing enteral feeding (Phase 2), and full enteral nutrition (Phase 3). In each phase, srSO_2_ and SCOR measurements obtained during and after feeding were compared to baseline (pre-feeding) values.

In Phase 1, srSO_2_ and SCOR measurements showed no significant differences before, during, or after feeding between Group 1 and Group 2 (*p* > 0.05) (Table 2). In Phase 2 and Phase 3, an increase in srSO_2_ and SCOR levels was observed in both groups, with a slightly greater increase in Group 2; however, these findings did not reach statistical significance (Phase 2 srSO_2_ *p* = 0.07, 0.08; SCOR *p* = 0.06, *p* = 0.06; Phase 3 srSO_2_ 0.06, 0.06, SCOR 0.07, 0.08) (Table 2). Furthermore, percentage changes in srSO_2_ and SCOR from baseline during and after feeding were evaluated in both groups, and these changes were significantly higher in the rapid advancement group (Table 2).

### 3.2. Subgroup Analysis of srSO_2_ and SCOR According to Feeding Tolerance vs. Feeding Intolerance in Groups 1 and 2

All infants in the study were subgrouped according to feeding tolerance (feeding-tolerant vs. feeding-intolerant), and srSO_2_ and SCOR values were analyzed accordingly. srSO_2_ and SCOR calculations were performed by adjusting remaining factors as null parameters including feeding tolerance, alterations during feeding period (before, during and after), feeding phases, feeding volume (slow/rapid advancement), gestational age, and birth weight.

### 3.3. srSO_2_ and SCOR Changes Across Feeding Phases in Groups 1 and 2 Based on Feeding Tolerance vs. Feeding Intolerance

**Figure 3:**
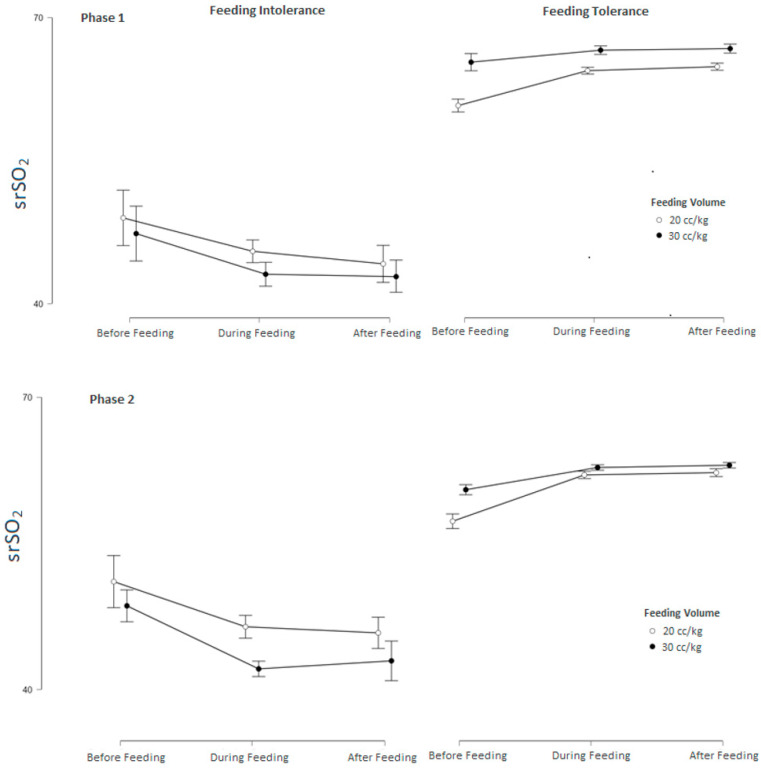

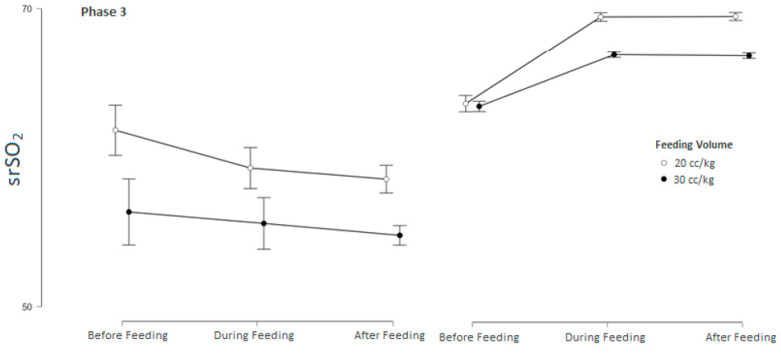

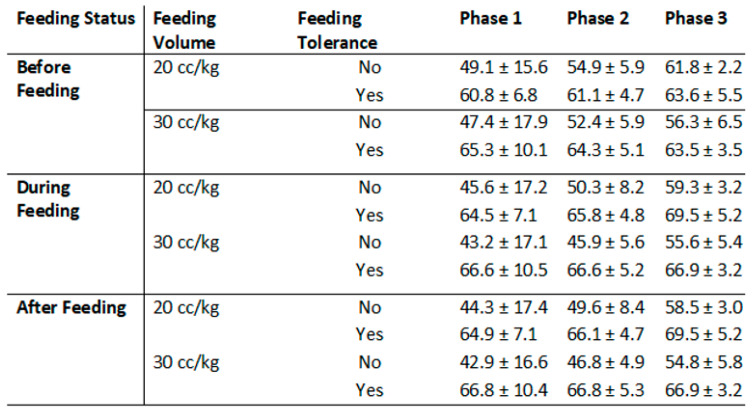
Changes and mean values of srSO_2_ measurements before (baseline), during, and after feeding across Phases 1–3 in feeding-tolerant vs. feeding-intolerant groups for groups 1 and 2.

**Figure 4:**
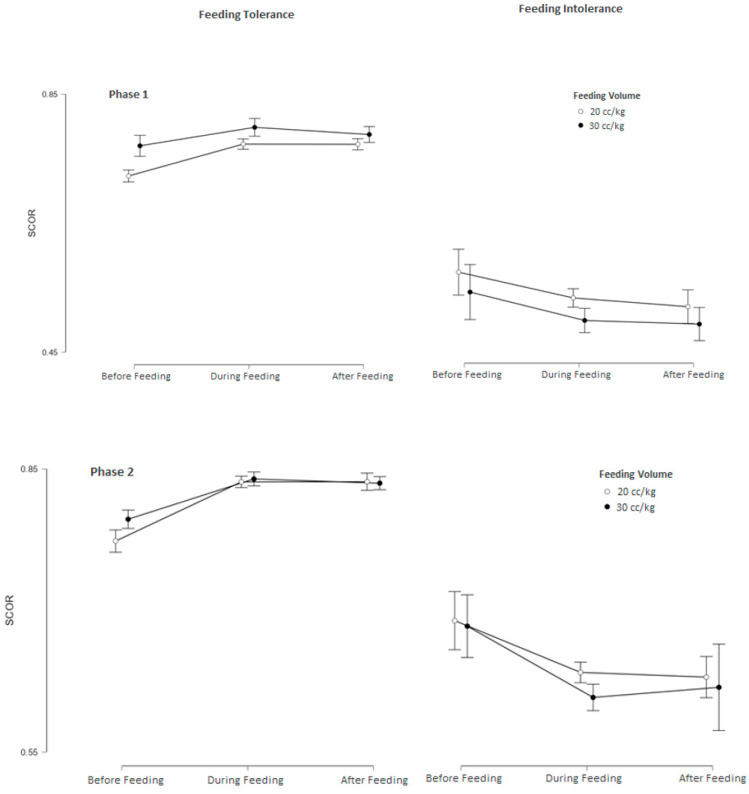

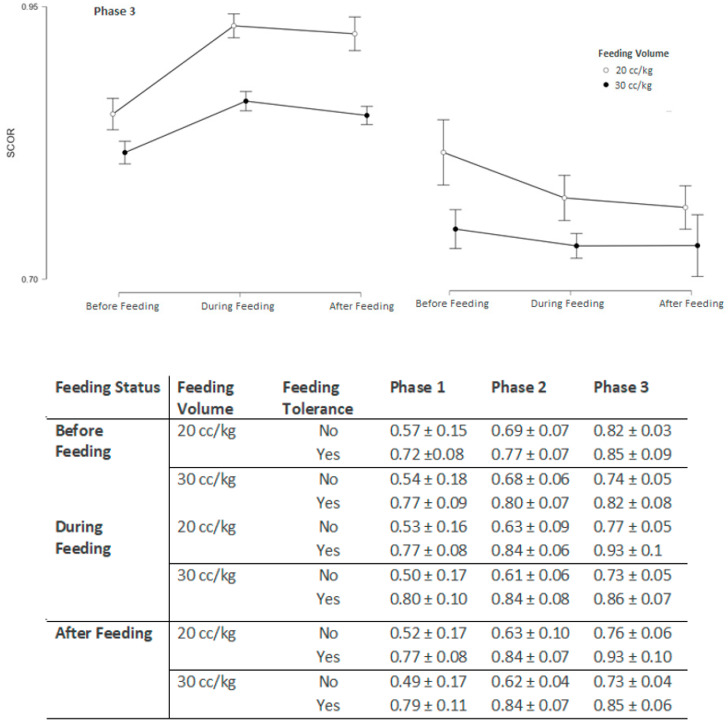
Changes and mean values of SCOR measurements before (baseline), during, and after feeding across Phases 1–3 in feeding-tolerant vs. feeding-intolerant groups for groups 1 and 2.

Feeding tolerance affected all measurements with extremely strong evidence (BF10 > 100, *p* < 0.001) compared to feeding intolerance in both groups. After adjustment, feeding intolerance continued to show a significantly decreasing effect on all measurements, with very strong (BF10 > 30) and extremely strong (BF10 > 100, *p* < 0.01) evidence in both groups (Table 3) (Figure 3 and Figure 4).

### 3.4. Estimating Feeding Tolerance Using a Composite ROC-Based Chart

We built ROC analysis with GW, BW, srSO_2_, and SCOR results for feeding tolerance. The prominent ROC results are presented in Table 4a.

Based on these findings, cut off values were determined as 30 weeks for gestational age (GA), 1180 g for birth weight (BW), 52 for abdominal NIRS-derived srSO_2_, and 0.631 for SCOR, as these thresholds provided the best sensitivity. Each parameter below its respective cutoff value was assigned 1 point, indicating an unfavorable condition. Subsequently, a new ROC analysis was conducted to assess the performance of this scoring model. A total score of 2 points yielded the best performance, with 100% sensitivity and 90.9% specificity, whereas 3 points provided maximum specificity with an acceptable level of sensitivity for predicting feeding intolerance (Table 4b).

## 4. Discussion

In this study, we evaluated the effects of rapid and slow advancement of enteral feeding on intestinal oxygenation in preterm infants. We found that both feeding strategies increased intestinal oxygenation; although this increase was not statistically significant. Nevertheless, the percentage change in both intestinal oxygenation and SCOR from baseline to during and after feeding was significantly higher in the rapid advancement group, particularly during the advancement phase and at full enteral feeding. These findings suggest that rapid feeding advancement does not impair intestinal perfusion and may even promote a more pronounced physiological response.

In this study, we found that enteral feeding increases metabolic demand and enhances mesenteric blood flow through the superior mesenteric artery (SMA), resulting in postprandial hyperemia [16]. Fang et al. observed that increased SMA blood flow velocity was associated with improved early enteral feeding tolerance in preterm infants, a result that is consistent with the more pronounced increase demonstrated in the rapid advancement group [25]. Similarly, Dix et al. [26] demonstrated an increase in postprandial intestinal oxygenation among feeding-tolerant infants. In another study, da Costa [27] reported that SCOR levels increased during enteral feeding in preterm infants without evidence of mesenteric ischemia. These results demonstrated that intestinal oxygenation increased in infants receiving intermittent bolus feeding regardless of whether advancement was slow or rapid, with a relatively greater increase observed in the rapid advancement group. Moreover, the percentage change was statistically significantly higher in the rapid advancement group. These results suggest that rapid advancement of enteral feeding does not negatively affect intestinal oxygenation; on the contrary, it appears to enhance it and does not reduce intestinal oxygenation, which is an important indicator of feeding tolerance [28,29,30]. Previous studies have shown that there are no differences in mortality or NEC between slow and rapid advancement strategies; our results are consistent with these findings and further strengthen the evidence supporting the safety of rapid advancement in this context [31].

In our study, infants with feeding intolerance had significantly lower srSO_2_ and SCOR levels compared with those who tolerated feeding, confirming that impaired intestinal oxygenation plays a key role in feeding difficulties. The significantly lower gestational age and birth weight observed in infants with feeding intolerance suggest that gastrointestinal immaturity adversely affects intestinal oxygenation [28,29]. In this context, the absence of significant differences between the two groups in other clinical characteristics known to influence feeding further supports this finding. Dani et al. [32] also reported lower splanchnic oxygenation and SCOR in infants with feeding intolerance, and Corvaglia et al. [33] found reduced splanchnic oxygenation in preterm infants with feeding intolerance. However, neither of these studies specifically addressed the rate of feed advancement. In our study, we also found that splanchnic oxygenation and SCOR values were lower in infants with feeding intolerance, independent of the rate of feeding advancement.

An important contribution of this study is the ROC-based prediction model we developed using gestational age, birth weight, srSO_2_, and SCOR values. The suggested cutoff values (GA < 30 weeks, BW < 1180 g, srSO_2_ < 52, SCOR < 0.6) demonstrated excellent predictive power for feeding intolerance, with 100% sensitivity and 90.9% specificity when ≥2 criteria were met. This model may help clinicians identify high-risk infants early and guide individualized feeding strategies. In our cohort, no NEC was detected. Since feeding intolerance can be a precursor of NEC, identifying infants with feeding intolerance is crucial. Patel et al. [34] reported that intestinal oxygenation levels were lower in infants who developed NEC and that NEC was observed in those with StO_2_ ≤ 56. Similarly, Paleri et al. [35] demonstrated that NEC developed in preterm infants <28 weeks of gestation with intestinal oxygenation <30%. As our study included infants between 28 and 32 weeks of gestation, this may explain why NEC was not observed in our cohort. We suggest that intestinal oxygenation <52 and a lack of increase or a decrease after feeding may serve as early warning indicators for feeding intolerance or NEC. However, it should be acknowledged that the ROC analysis represents a secondary analysis of the data, and its findings should be interpreted with caution.

Our findings align with most reports indicating that rapid advancement of enteral feeding reduces the time to achieve full enteral feeding without increasing the risk of NEC or feeding intolerance. By demonstrating that rapid advancement does not adversely affect intestinal oxygenation, our study provides additional evidence supporting its safety and potential clinical benefit in preterm infants.

### Limitations

A major limitation of this study is the exclusion of preterm infants born at <28 weeks of gestational age, who are expected to have higher rates of feeding intolerance and NEC. Additionally, infants who were small for gestational age or had intrauterine growth restriction both conditions associated with increased feeding intolerance risk were not included in the study.

## 5. Conclusions

Intermittent bolus feeding in preterm infants increases intestinal oxygenation from baseline during and after enteral feeding, and this effect was found to be more pronounced with rapid advancement. No differences were observed in gastrointestinal adverse outcomes between slow and rapid advancement of enteral feeding. In infants with feeding intolerance, intestinal oxygenation remained lower than baseline throughout minimal enteral feeding and the advancement phase, and this reduction persisted after feeding. The findings of this study would be valuable if further investigated in future research involving smaller preterm infants.

## Figures and Tables

**Table 1:** Demographic and Clinical Features of Infants in Groups 1 and 2.

	Groups 1 (n: 30)	Groups 2 (n: 30)	*p*
Gestational age (weeks), mean ± SD	29.8 ± 1.3	29.6 ± 0.2	0.555
Birth weight (g), median (min–max)	1193 (1100–1426)	1405 (1199–1458)	0.190
Gender, (M) n (%)	16 (53)	14 (47)	1
Antenatal steroid, n (%)	17 (57)	18 (60)	1
APGAR at 5 min, median (min–max)	8 (7–9)	8 (7–8)	0.780
SNAPPE II, median (min–max)	0 (0–4.3)	0 (0–3.9)	0.553
Prolonged membrane rupture, n (%)	3 (10.0)	4 (13.3)	0.127
Chorioamnionitis, n (%)	0	1 (3.3)	1
Gestational hypertension, n (%)	7 (23.3)	4 (13.3)	0.313
Preeclampsia, n (%)	10 (33.3)	6 (20.0)	0.237
Caffeine, n (%)	21 (70.0)	16 (53.3)	0.158
Surfactant, n (%)	19 (63.3)	16 (56.6)	0.132
Invasive mechanical ventilation duration (days), median (min–max)	2 (0–5.3)	1 (0–3.3)	0.15
Non-invasive mechanical ventilation duration (days), median (min–max)	5 (3–13)	6 (2.8–12.5)	0.889
Stage ≥ 2 NEC, n (%)	0	0	N/A *
Late onset sepsis, n (%)	5 (16.7)	6 (20.0)	0.217
HsPDA, n (%)	5 (16.7)	4 (13.3)	0.716
Stage 3 ≥ ROP, n (%)	0	0	N/A
Stage 2 ≥ IVH, n (%)	0	0	N/A
Exclusive breastfeeding, n (%)	21 (70.0)	23 (76.6)	0.771
Minimal enteral feeding duration (days), mean ± SD	2.8 ± 1.7	2.3 ± 0.9	0.184
Feeding intolerance, n (%)	8 (26.6)	6 (20.0)	0.192
Full enteral feeding transition duration (days), median (min–max)	9 (7.8–11.1)	7 (6.3–9.2)	0.005
Hospital stay (days), mean ± SD	41.2 ± 18.5	40.9 ± 19.9	0.955
Mortality, n (%)	0	0	N/A

* N/A: Not Applicable.

**Table 2:** Comparison of srSO_2_ and SCOR Values and Percentage Changes Between Slow and Rapid Advancement Groups in Feeding Phases.

	Groups 1	Groups 2	*p*
Phase 1			
Pre-feeding (baseline) srSO_2_	57.51 ± 11.23	59.81 ± 12.82	0.13
During-feeding srSO_2_	59.14 ± 13.72	60.35 ± 14.06	0.26
After-feeding srSO_2_	59.08 ± 14.35	60.42 ± 14.07	0.25
Percentage Change (baseline to during-after feeding)	2.78 ± 0.86	2.54 ± 0.92	0.73
Pre-feeding (baseline) SCOR	0.71 ± 0.13	0.72 ± 0.12	0.12
During-feeding SCOR	0.75 ± 0.25	0.76 ± 0.27	0.23
After-feeding SCOR	0.76 ± 0.28	0.77 ± 0.24	0.29
Percentage Change (baseline to during-after feeding)	2.92 ± 1.34	3.53 ± 1.42	0.9
Phase 2			
Pre-feeding (baseline) srSO_2_	59.34 ± 5.74	60.61 ± 6.62	0.42
During-feeding srSO_2_	62.45 ± 9.23	66.75 ± 9.07	0.07
After-feeding srSO_2_	62.83 ± 9.53	66.92 ± 8.85	0.08
Percentage Change (baseline to during-after feeding)	5.25 ± 2.16	10.14 ± 3.24	0.03
Pre-feeding (baseline) SCOR	0.74 ± 0.13	0.73 ± 0.12	0.96
During-feeding SCOR	0.81 ± 0.11	0.85 ± 0.11	0.06
After-feeding SCOR	0.81 ± 0.12	0.87 ± 0.12	0.06
Percentage Change (baseline to during-after feeding)	3.93 ± 2.34	11.97 ± 3.26	0.02
Phase 3			
Pre-feeding (baseline) srSO_2_	62.26 ± 4.93	62.74 ± 4.46	0.71
During-feeding srSO_2_	69.94 ± 6.63	72.75 ± 5.07	0.06
After-feeding srSO_2_	69.75 ± 6.87	72.84 ± 5.23	0.06
Percentage Change (baseline to during-after feeding)	12.42 ± 3.45	15.91 ± 4.85	0.02
Pre-feeding (baseline) SCOR	0.70 ± 0.08	0.74 ± 0.07	0.77
During-feeding SCOR	0.84 ± 0.11	0.86 ± 0.08	0.07
After-feeding SCOR	0.83 ± 0.12	0.88 ± 0.07	0.08
Percentage Change (baseline to during-after feeding)	13.34 ± 5.21	16.04 ± 5.41	0.04

**Table 3:** Comparison of srSO_2_ and SCOR Values Between Infants With and Without Feeding Intolerance in Feeding Phases.

	Feeding Intolerance (n: 14)	Feeding Tolerance (n: 46)	*p*
Phase 1			
Pre-feeding (baseline) srSO_2_	48.53 ± 15.64	63.34 ± 9.04	0.008
During-feeding srSO_2_	44.83 ± 16.46	65.65 ± 9.12	0.001
After-feeding srSO_2_	43.84 ± 16.42	65.91 ± 9.06	<0.001
Pre-feeding (baseline) SCOR	0.57 ± 0.15	0.72 ± 0.08	<0.001
During-feeding SCOR	0.54 ± 0.18	0.77 ± 0.09	<0.001
After-feeding SCOR	0.53 ± 0.165	0.77 ± 0.10	<0.001
Phase 2			
Pre-feeding (baseline) srSO_2_	54.15 ± 5.73	62.91 ± 5.17	<0.001
During-feeding srSO_2_	48.83 ± 7.52	66.34 ± 5.08	<0.001
After-feeding srSO_2_	48.71 ± 7.35	66.52 ± 5.07	<0.001
Pre-feeding (baseline) SCOR	0.69 ± 0.07	0.77 ± 0.07	<0.001
During-feeding SCOR	0.68 ± 0.06	0.80 ± 0.07	<0.001
After-feeding SCOR	0.63 ± 0.09	0.84 ± 0.06	<0.001
Phase 3			
Pre-feeding (baseline) srSO_2_	60.25 ± 4.42	63.51 ± 4.53	0.035
During-feeding srSO_2_	58.23 ± 4.15	68.16 ± 4.47	<0.001
After-feeding srSO_2_	57.43 ± 4.17	68.13 ± 4.45	<0.001
Pre-feeding (baseline) SCOR	0.82 ± 0.03	0.85 ± 0.084	0.165
During-feeding SCOR	0.74 ± 0.05	0.82 ± 0.09	<0.001
After-feeding SCOR	0.77 ± 0.05	0.88 ± 0.09	<0.001

**Table 4:** (a) Estimation of Feeding Intolerance Based on a Composite Metric Score. (b) srSO_2_, gestational age, and SCOR values as the best predictors of feeding intolerance.

(a)											
Cutpoint	Sensitivity (%)		Specificity (%)		PPV (%)		NPV (%)		AUC		Metric Score
0	100%		0%		21.43%		NaN%		0.989		1
1	100%		54.55%		37.50%		100%		0.989		1.55
2	100%		90.91%		75%		100%		0.989		1.91
3	75%		100%		100%		93.62%		0.989		1.75
4	58.33%		100%		100%		89.80%		0.989		1.58
(b)											
*srSO_2_ Baseline*											
Cutpoint		Sensitivity (%)		Specificity (%)		PPV (%)		NPV (%)		AUC	
52		100%		66.67%		91.67%		100%		0.805	
52.5		97.73%		66.67%		91.49%		88.89%		0.805	
55		88.64%		75%		92.86%		64.29%		0.805	
*BW*											
Cutpoint		Sensitivity (%)		Specificity (%)		PPV (%)		NPV (%)		AUC	
1180		84.09%		91.67%		97.37%		61.11%		0.918	
1195		79.55%		100%		100%		57.14%		0.918	
1205		77.27%		100%		100%		54.55%		0.918	
1245		75%		100%		100%		52.17%		0.918	
*GW*											
Cutpoint		Sensitivity (%)		Specificity (%)		PPV (%)		NPV (%)		AUC	
30		63.64%		100%		100%		42.86%		0.811	
*SCOR Baseline*											
Cutpoint		Sensitivity (%)		Specificity (%)		PPV (%)		NPV (%)		AUC	
0.6318		0.9773		0.7500		0.9348		0.9000		0.8470	
0.6322		0.9545		0.7500		0.9333		0.8182		0.8470	
0.6353		0.9318		0.7500		0.9318		0.7500		0.8470	

## Data Availability

The data supporting the findings of this study are not publicly available due to privacy and ethical restrictions involving patient confidentiality. Data may be available from the corresponding author upon reasonable request and with permission from the relevant institutional review boards.

## Abbreviations

The following abbreviations are used in this manuscript:

NIRS	Near-infrared spectroscopy
SMA	Superior mesenteric artery
NEC	Necrotizing enterocolitis
srSO_2_	Splanchnic oxygenation
